# Molecular epidemiology of *Staphylococcus aureus* in pediatric cystic fibrosis patients: a single-center study

**DOI:** 10.3389/fcimb.2026.1808334

**Published:** 2026-05-28

**Authors:** Katarzyna Garbacz, Lidia Piechowicz, Aneta Mroczkowska, Joanna Empel, Jacek Międzobrodzki, Maja Kosecka-Strojek

**Affiliations:** 1 Department of Oral Microbiology, Medical Faculty, Medical University of Gdansk, Gdansk, Poland; 2 Department of Medical Microbiology, Medical Faculty, Medical University of Gdansk, Gdansk, Poland; 3 Department of Epidemiology and Clinical Microbiology, National Medicines Institute, Warsaw, Poland; 4 Department of Microbiology, Faculty of Biochemistry, Biophysics and Biotechnology, Jagiellonian University in Krakow, Krakow, Poland

**Keywords:** cystic fibrosis, MRSA, pediatric patients, *spa* typing, *Staphylococcus aureus*

## Abstract

**Introduction:**

*Staphylococcus aureus* is a key respiratory pathogen in children with cystic fibrosis (CF). This study aimed to evaluate the prevalence and relatedness of *spa* types of *S. aureus* respiratory isolates from pediatric CF patients, grouped by age, and to assess their antimicrobial resistance.

**Methods:**

A total of 165 *S. aureus* isolated from 82 patients from single-center and diagnosed with cystic fibrosis over a three-year period were analyzed. Molecular typing (*spa* typing and SCC*mec* typing) and antimicrobial susceptibility testing were performed.

**Results:**

Sixty-four *spa* types were identified, including five novel types, and the isolates were assigned to 11 multilocus sequence typing (MLST) clonal complexes, most frequently CC30, CC22, CC97, CC45, and CC15. In 67.1% of patients, only a single *spa* type was detected. In cases where multiple types were isolated from a single patient, 85.2% of the isolates were not clonally related. Macrolide and lincosamide resistance was common, particularly among adolescents aged 12–18 years, at rates of 67.4% and 60.9%, respectively. Resistance to fluoroquinolones, aminoglycosides, tetracyclines, and co-trimoxazole did not exceed 15%. MRSA was detected in 10.7% of patients and exclusively among those with monoclonal colonization.

**Conclusion:**

The finding indicates that *S. aureus* colonization in pediatric CF patients is highly diverse and primarily individualized, with limited evidence of clonal transmission. Given the high prevalence of macrolide resistance-particularly among adolescents-long-term macrolide therapy should be carefully monitored, with periodic microbiological surveillance to mitigate resistance selection.

## 1 Introduction

*Staphylococcus aureus* is one of the most prevalent respiratory pathogens in pediatric cystic fibrosis (CF) patients and contributes to chronic airway colonization. It is isolated in up to 80% of children, usually shortly after diagnosis (Elborn, 2016; Zolin et al., 2022). In some cases, staphylococcal colonization can persist throughout the patient’s life. Respiratory colonization with *S. aureus* strains is particularly common during the first decade of life and significantly contributes to morbidity and, in some cases, mortality in children with CF. *S. aureus* strains may negatively affect lung function in cystic fibrosis patients, leading to a high risk of life-threatening infections (Elborn, 2016; Zolin et al., 2022). Methicillin-resistant *S. aureus* (MRSA) infections are becoming an increasingly serious problem for individuals with CF, often resulting in more severe lung disease. They can lead to deterioration in lung function and negatively impact other growth and development parameters in children. In addition, patients with MRSA infections may experience an increased frequency of antibiotic use and hospitalization. Chronic MRSA infection may also be associated with severe, life-threatening complications, particularly in patients undergoing immunosuppressive therapy following organ transplantation (Chmiel et al., 2014; Esposito et al., 2019).

Long-term antibiotic therapy in children with CF exerts significant selective pressure on bacteria. This pressure can lead to the adaptation of *S. aureus*, resulting in the development of antibiotic-resistant clones that can survive in the respiratory tract despite treatment (Chmiel et al., 2014). However, there is still limited knowledge about the clones that colonize the respiratory tract of CF patients and how these clones change over time.

*spa* typing is one of the most used molecular tools for analyzing the clonality of *S. aureus* strains. It involves sequencing the polymorphic region of protein A (Variable Number of Tandem Repeats, VNTR region). Furthermore, recent reports have shown that *spa* typing results are consistent with the more advanced and costly whole genome sequencing (WGS) (Rumpf et al., 2021).

This study aimed to assess the prevalence, genetic relatedness (*spa* typing), and antimicrobial resistance patterns of *S. aureus* respiratory isolates collected from 82 pediatric cystic fibrosis patients, over a three-year period.

## 2 Methods

### 2.1 *S. aureus* isolates

The retrospective study included material from 82 CF pediatric patients aged 1 month to 18 years (mean age: 7.8 years; median age: 8.5 years), treated at the Outpatient Cystic Fibrosis Clinic at “Polanki” Children’s Hospital in Gdansk, over a three-year period (between 2012 and 2014). Children were divided into four age groups: 1. infants - 0–1 year (n=11), 2. toddlers - 2–5 years (n=22), 3. school-age children - 6–11 years (n=28), and 4. adolescents - 12–18 years (n=21). The number of samples collected (throat swabs, sputum, and bronchoalveolar lavage) varied depending on the treatment course, ranging from at least one per patient to two or even seven. All samples were obtained as part of routine laboratory diagnostic procedures and not collected specifically for the purposes of this study. The materials were subcultured on Columbia blood agar and Chapman agar (bioMérieux, Marcy l’Étoile, France) and incubated aerobically at 37°C for 18 to 24 hours. *S. aureus* isolates were identified according to standard microbiological diagnostic procedures and confirmed by the detection of the thermostable nuclease gene (*nuc*SA) (Baron et al., 2004; Tille, 2013). The isolates were stored at -80°C in Trypticase Soy Broth (Oxoid, UK) supplemented with 15% glycerol.

### 2.2 Preparation of bacterial DNA

Total DNA of *S. aureus* isolates was purified using Genomic Mini DNA kit (A&A Biotechnology, Poland), in line with the manufacturer’s instructions.

### 2.3 *spa* typing

*spa* typing was performed according to Harmsen et al. (2003). The resulting sequences were analyzed using Ridom StaphType software v.2.1.1 (Ridom GmbH, Würzburg, Germany) and compared against the Ridom SpaServer database (https://spaserver.ridom.de/). *spa* types were clustered into *spa*-clonal complexes (*spa*-CCs) using the Based Upon Repeat Pattern (BURP) algorithm implemented in Ridom SeqSphere+ software (Ridom GmbH, Münster, Germany) using the following parameters: “exclude *spa* types shorter than five repeats” and “cluster *spa* types into the same group if cost distances are less than four” (Mellmann et al., 2007). For *spa*-CCs without a defined founder, lowercase letters were assigned to designate the cluster name. Based on the obtained *spa* types, the Ridom SpaServer database (http://spaserver.ridom.de/), and published literature, the assignment of the studied isolates to MLST clonal complexes (hereinafter referred to as CCs) could be predicted.

### 2.4 Antimicrobial susceptibility testing

Antimicrobial susceptibility of *S. aureus* isolates to antimicrobial agents was determined by disk diffusion and interpreted according to the CLSI document no. M100-S22 (Clinical and Laboratory Standards Institute, 2012). The following drugs were used for the test: penicillin, oxacillin, erythromycin, roxithromycin, clindamycin, ciprofloxacin, ofloxacin, tetracycline, amikacin, gentamicin, fusidic acid (Jones et al., 2010), co-trimoxazole (sulfamethoxazole/trimethoprim), chloramphenicol (all from Becton Dickinson, Germany), and mupirocin (Oxoid, UK). Multidrug resistance was defined as resistance to antimicrobial agents from at least three various classes (MDR). For isolates identified as resistant to erythromycin but susceptible to clindamycin, the D-test was performed to detect inducible clindamycin resistance. The minimal inhibitory concentration (MIC) of vancomycin was determined using E-tests, in accordance with the manufacturer’s instructions (AB Biodisc, Sweden). The reference strain *S. aureus* ATCC 29213 was used for quality control.

### 2.5 Detection of methicillin-resistance and determination of SCC*mec* cassette type

The isolates were screened for their resistance to oxacillin based on the growth of blue colonies in the selective medium (ORSAB, Oxoid, UK). Suspected methicillin-resistant *S. aureus* (MRSA) isolates were further examined for the presence of the *mec*A gene, as described elsewhere (Murakami et al., 1991). Each PCR contained *mec*A-positive (*S. aureus* ATCC 43300) and *mec*A-negative (*S. aureus* ATCC 29213) strains as controls. All *mec*A-negative *S. aureus* isolates able to grow on ORSAB plates were tested for the carriage of the *mec*C gene using primers described by Cuny et al. (2011). Typing of the staphylococcal chromosomal cassette *mec* (SCC*mec*) was carried out as described previously, Milheirico et al (Milheiriço et al., 2007; Wolska-Gębarzewska et al., 2024).

### 2.6 Statistical analysis

All calculations were performed with the Statistica package (StatSoft, Tulsa, OK, USA), with a significance threshold set at a *p*-value 0.05. The significance of differences percentages of antibiotic-resistant isolates between age groups was verified with Pearson’s chi-squared test or Fisher’s exact test.

## 3 Results

### 3.1 *S. aureus* isolates distribution

A total of 165 *S. aureus* isolates were collected from 82 pediatric patients diagnosed with cystic fibrosis over a three-year period. Most patients (39/82; 47.5%) had a single isolate cultured. Two isolates were obtained from each of 18 patients (22%), three isolates from each of 17 patients (20.7%), four isolates from each of three patients, five isolates from each of four patients, and seven isolates from one patient. The number of isolates per patient and time point was 2.01 on average (median: 2; range: 1–7 isolates). Overall, 79 isolates originated from throat swabs, 69 from sputum, and 17 from bronchoalveolar lavage.

All collected isolates were stratified according to the children’s age groups: under 1 years old (group I, n=16), 2 to 5 years old (group II, n=39), above 6 up to 11 years old (group III, n=64), and above 12 to 18 years old (group IV, n=46) (**Table 1**).

**Table 1:** Distribution of pediatric CF patients and *S. aureus* isolates by age group.

Age group	Number of patients (%)	Number of isolates (%)
0–1 year	11 (13.4%)	16 (9.7%)
2–5 years	22 (26.8%)	39 (23.6%)
6–11 years	28 (34.1%)	64 (38.8%)
12–18 years	21 (25.6%)	46 (27.9%)
Total	82	165

### 3.2 *spa* typing and clustering

A total of 64 distinct *spa* types were identified among the 165 *S. aureus* isolates examined. The most prevalent *spa* types were t091 (n=10, 6.1%), t700 (n=10, 6.1%), t015 (n=8, 4.8%), t005 (n=7, 4.2%), t078 (n=6, 3.6%), and t571 (n=6, 3.6%), collectively representing approximately one-third of all isolates (n=47, 28.5%). In addition, five new *spa* types: t14287 (n=1), t14288 (n=2), t14289 (n=5), t14290 (n=1), and t21921 (n=2), were identified and registered in the international Ridom SpaServer database.

**Figure 1:**
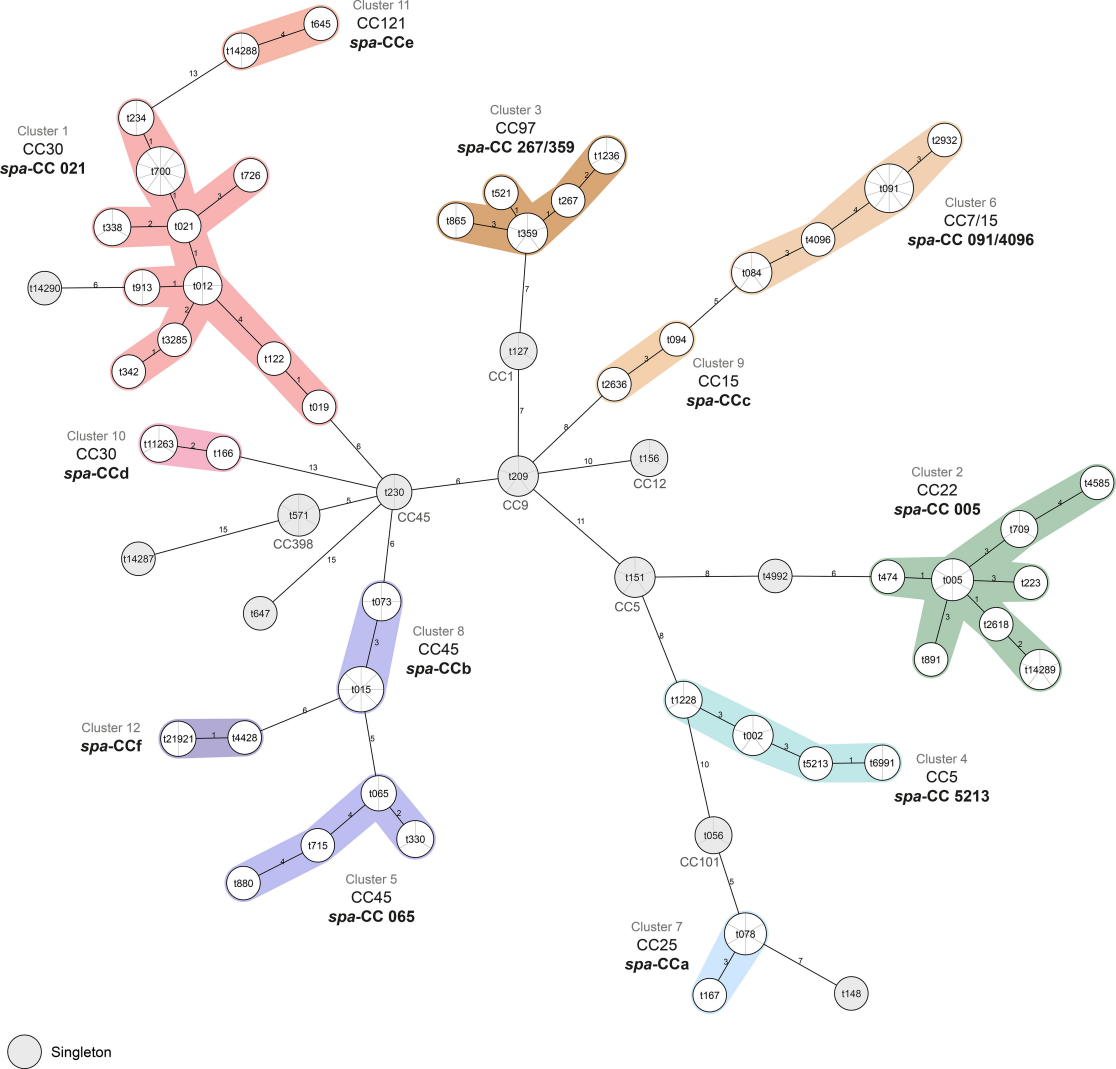
A minimum spanning tree (MST), created using Ridom SeqSphere+ software, based on *spa* type profiles of 157 *S. aureus* isolates recovered from CF patients with *spa*-CCs/CCs indicated.

The identified *spa* types were clustered into 12 *spa*-CCs. Most isolates belonged to the following five clusters: *spa-*CC 021 (n=27, 16.4%), *spa*-CC 005 (n=20, 12.1%), *spa*-CC 091/4096 (n=17, 10.3%), *spa*-CC 267/359 (n=12, 7.3%), and *spa*-CC 5213 (n=11, 6.6%). Among all isolates, 32 (19.4%) were classified as singletons, representing 12 distinct *spa* types, whereas eight (4.8%) isolates, corresponding to *spa* types t026, t362, t693, and t748, were excluded from BURP analysis (**Figure 1**).

### 3.3 *spa* type relatedness

Among the 82 patients included in the study, *S. aureus* isolates representing a single *spa* type were identified in most patients (55/82; 67.1%). Two distinct *spa* types were detected in each of 24 patients (29.3%), while three different *spa* types were found only in each of three patients (3.6%) (**Supplementary Tables 1**, **2**).

#### 3.3.1 One patient – one *spa* type

**Figure 2:**
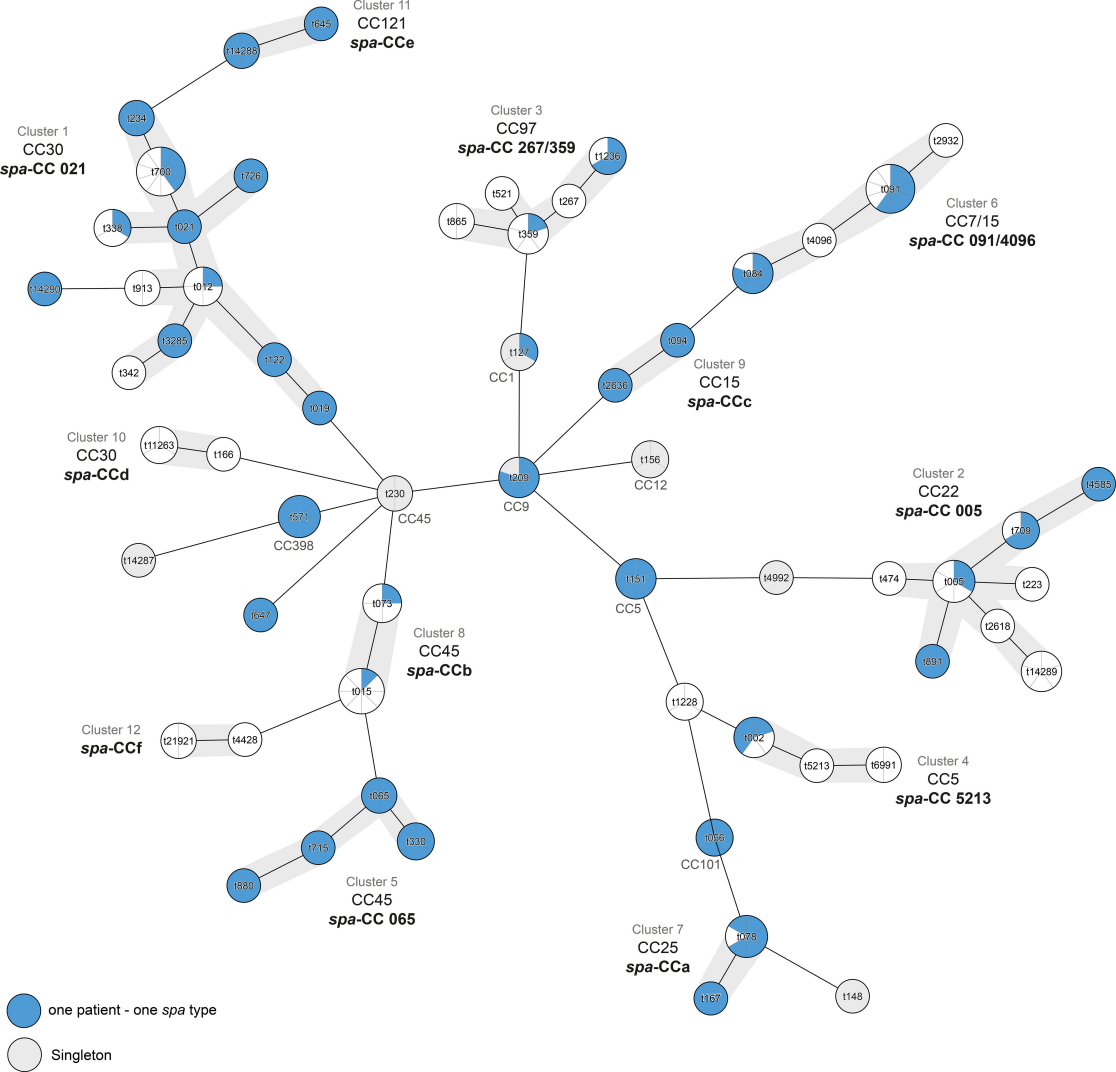
A minimum spanning tree (MST), created from *spa* type profiles of 157 *S. aureus* isolates recovered from CF patients. The *spa* types from the group „one patient – one *spa* type” are indicated within *spa*-CCs/CCs clusters.

In most cases involving 39 patients, only one isolate was recovered throughout the entire treatment period. Two isolates were recovered from eight patients, three isolates from six patients, and five isolates from two patients, all belonging to the same *spa* type. A total of 41 *spa* types (64%) were identified among these patients, with t571 (n=6) and t091 (n=6) being the most prevalent. All t571 isolates (from two patients) belonging to CC398 were found exclusively within this group. In total, 83 isolates obtained from 24 patients were distributed across most clusters (**Figure 2**). Notably, *spa*-CC 065, *spa*-CCc, and *spa*-CCe each included 100% of the isolates within their respective clusters (**Table 2**).

**Table 2:** Distribution of *spa* types and *spa*-CCs in patients carrying single *spa* types.

*Spa*-CC	*Spa* type (no. of isolates per patient)	% of each *spa*-CC
*spa*-CC 021 (CC30)	t012 (1), t019 (1), t021 (1), t122 (1), t234 (2), t338 (1), t700 (1), t700 (1), t700 (2) t726 (1), t3285 (1)	48.1%
*spa*-CC 005 (CC22)	t005 (1), t005 (2), t709 (2), t891 (1), t4585 (1)	35%
*spa*-CC 267/359 (CC97)	t359 (1), t1236 (1), t1236 (1)	25%
*spa*-CC 5213 (CC5)	t002 (1), t002 (2)	27.3%
*spa*-CC 065 (CC45)	t065 (1), t065 (1), t330 (3), t715 (1), t880 (1)	100%
*spa*-CC 091/4096 (CC7/15)	t084 (1), t084 (3), t091 (1), t091 (1), t091 (1), t091 (3)	58.8%
*spa*-CCa (CC25)	t078 (5), t167 (1)	85.7%
*spa*-CCb (CC45)	t015 (1), t073 (1)	16.7%
*spa*-CCc (CC15)	t094 (1), t2636 (1)	100%
*spa*-CCe (CC121)	t645 (1), t14288 (2)	100%
singleton	t056 (1), t056 (2), t127 (1), t151 (5), t209 (1), t209 (3), t230 (1), t571 (3), t571 (3), t647 (1), t14290 (1)	68.7%
excluded	t026 (1), t362 (2), t693 (1), t693 (1)	62.6%

#### 3.3.2 One patient – two *spa* types

The distribution of the identified *spa* types and the corresponding number of isolates per patient is summarized in **Table 3**. In this group of patients, the most frequently isolated *spa* types were t700 (5 patients), t005 (4 patients), and t015 (4 patients). Eight clusters and six singletons were identified. Unrelated types were observed in 87.5% of cases involving 21 patients. The most common combination of isolates was *spa*-CC021 and *spa*-CC005, representing CC30 and CC22, respectively. Isolates of two different but related *spa* types (belonging to the same CCs) were recovered from only three patients. The first was patient 31, from whom three isolates were detected: one t4428 and two t21921, both of which *spa* types grouped into *spa*-CCf. This cluster comprised exclusively isolates derived from this patient. The second was patient 35, from whom two isolates, t005 and t474, were recovered and grouped into *spa*-CC 005. The third was patient 49, who carried one isolate of t5213 and two isolates of t6991, grouped into *spa*-CC 5213 (CC5). Interestingly, among the eight patients with isolates of *spa*-CC 021, three also had *spa*-CC 005 detected (patients P.1, P.10, and P.15). Isolates belonging to *spa*-CCb and *spa*-CC 5213 constituted the majority within the described *spa*-CCs, accounting for 83.3% and 72.7%, respectively. In this group of patients, isolates clustered within *spa*-CC 267/359 accounted for 50%. Furthermore, each of the five main *spa*-CCs were represented by four different *spa* types.

**Table:** Table 3 Distribution of *spa* types and *spa*-CCs in patients carrying two *spa* types.

*Spa-CC*	*Spa* type	Patient ID (no. of isolates)	% of each *spa-CC*
*spa*-CC 021 (CC30)	t012	P.1 (3)	44.4%
	t342	P.10 (1)	
	t338	P.15 (2)	
	t700	P.2 (1), P.14 (1), P.22 (1), P.28 (1), P.37 (2)	
*spa*-CC 005 (CC22)	t005	P.1 (1), P.15 (1), P.32 (1), P.35 (1)	35%
	t223	P.10 (1)	
	t474	P.35 (1)	
	t709	P.64 (1)	
*spa*-CC 267/359 (CC97)	t359	P.9 (1), P.32 (1)	50%
	t521	P.41 (1)	
	t865	P.60 (2)	
	t1236	P.26 (1)	
*spa*-CC 5213 (CC5)	t002	P.2 (2)	72.7%
	t1228	P.4 (1), P.26 (2)	
	t5213	P.49 (1)	
	t6991	P.49 (2)	
*spa*-CC 091/4096 (CC7/15)	t084	P.40 (1)	41.2%
	t091	P.21 (1), P.25 (2), P.60 (1)	
	t2932	P.41 (1)	
	t4096	P.28 (1)	
*spa*-CCa (CC25)	t078	P.64 (1)	14.3%
*spa*-CCb (CC45)	t015	P.9 (1), P.21 (3), P.22 (1), P.24 (2)	83.3%
	t073	P.40 (3)	
*spa*-CCf	t4428	P.31 (1)	100%
	t21921	P.31 (2)	
singleton	t127	P.4 (2)	28.1%
	t148	P.25 (1)	
	t156	P.11 (2), P.14 (1)	
	t230	P.38 (1)	
	t4992	P.24 (1)	
	t14287	P.11 (1)	
excluded	t748	P.37 (1), P.38 (1)	25%

#### 3.3.3 One patient – three *spa* types

In total, 17 isolates collected from three patients, each colonized with three distinct *spa* types, were assigned to four *spa*-CCs: *spa*-CC 021, *spa*-CC 005, *spa*-CC 267/359, and *spa*-CCd (**Figure 1**). Two *S. aureus* isolates assigned to CC9 were not classified into *spa*-CCs; these included *spa* type t209, identified as a singleton, and t693, which was excluded from the *spa*-CC analysis based on the criteria used. The isolates from each patient were assigned into the following CCs and *spa* types: patient 3, CC30 (t166, t913) and CC97 (t359), patient 18, CC9 (t693), CC30 (t11263), and CC97 (t267), and patient 20, CC9 (t209) and CC22 (t2618, t14289).

### 3.4 Antimicrobial susceptibility

The susceptibility testing of *S. aureus* isolates revealed the following resistance rates for tested antimicrobial agents: penicillin 94.5%, erythromycin 54.5%, roxithromycin 54.5%, clindamycin 49.1%, ciprofloxacin 14.5%, ofloxacin 14.5%, gentamicin 10.3%, netilmicin 10.3%, amikacin 9.7%, tetracycline 7.9%, oxacillin 5.5%, and co-trimoxazole 2.4%. All isolates were susceptible to vancomycin, linezolid, fusidic acid, mupirocin, and chloramphenicol. The D-test identified inducible clindamycin resistance (iMLSB) in 11.5% of isolates.

**Figure 3:**
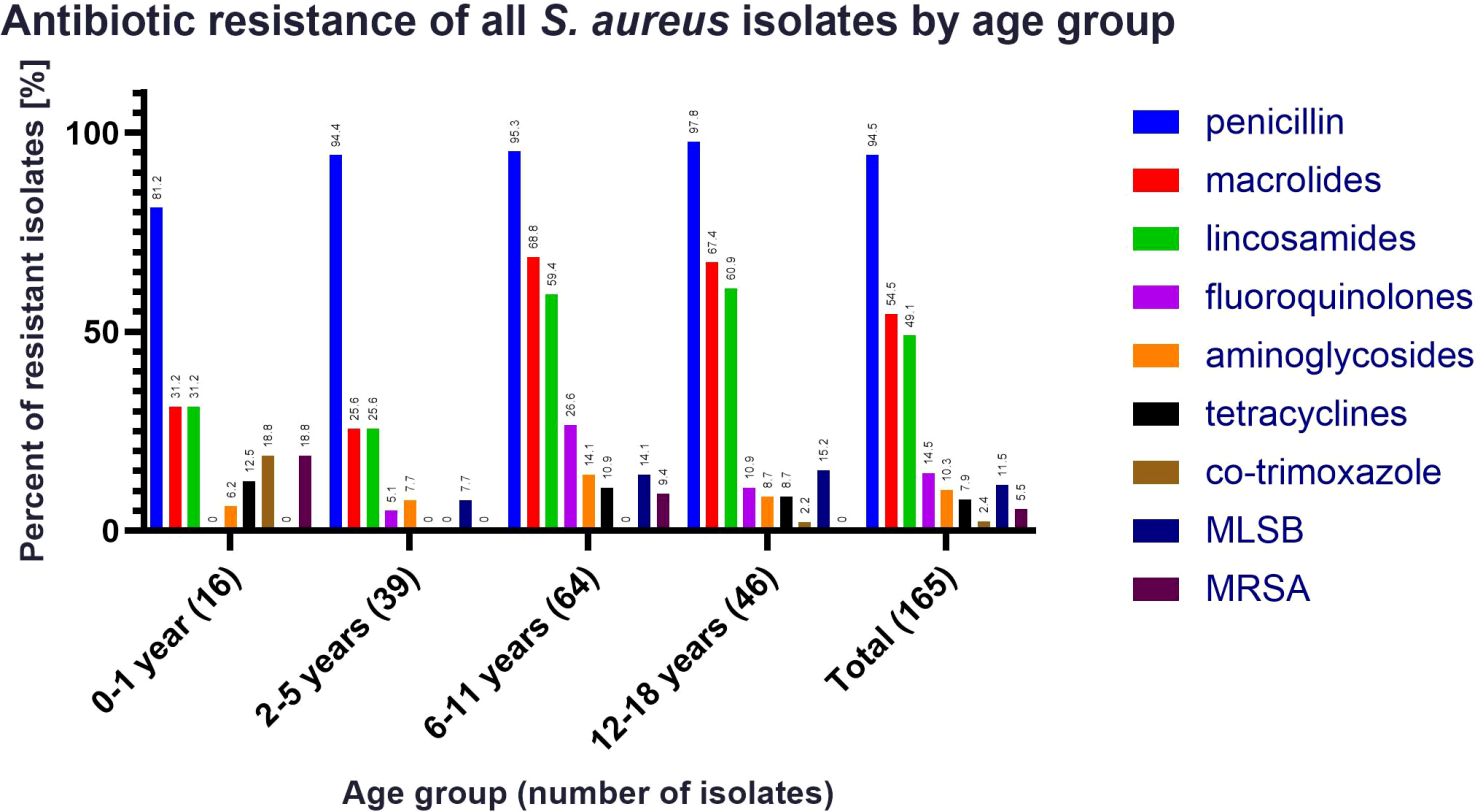
Antibiotic resistance of all *S. aureus* isolates by age group. The graph was created using GraphPad Prism version 10.6.0 for Windows, GraphPad Software, Boston, Massachusetts USA, www.graphpad.com.

The highest resistance rates were observed for penicillin across all age groups, reaching 94.5%. Overall, there was an increasing trend in antimicrobial resistance with advancing age. The most significant increases in resistance were observed for macrolides and lincosamides, with resistance in the 12–18-year age group reaching 60% for both classes of antibiotics (p < 0.05). Resistance to fluoroquinolones, aminoglycosides, tetracyclines, and co-trimoxazole remained notably lower across all age groups, not exceeding 15% (**Figure 3**; **Supplementary Tables 3**, **4**).

### 3.5 Methicillin-resistant *S. aureus* isolates

Nine (5.5%) *S. aureus* isolates resistant to oxacillin were identified as MRSA. These isolates originated from three patients: one infant (three isolates from P.51) and two school-aged children (six isolates from P.12 and P.19). All MRSA isolates were multidrug-resistant, exhibiting resistance to macrolides, lincosamides, fluoroquinolones, aminoglycosides, and co-trimoxazole. The MRSA isolates were *mec*A-positive and represented three distinct lineages: CC5-t151-SCC*mec* II, CC45-t073-SCC*mec* II, and CC398-t571-SCC*mec* V. Importantly, all MRSA isolates were exclusively derived from patients colonized by isolates of a single *spa* type, accounting for 10.7% of the cases.

## 4 Discussion

For years, *spa* typing has been an invaluable tool in monitoring *S. aureus* infections in patients with cystic fibrosis, particularly in assessing relatedness of strains and potential sources of transmission. In our study, analysis of *S. aureus* isolates from CF children revealed pronounced genetic diversity, with no evidence of a dominant clone. Identical *spa* types were detected only sporadically among different patients, suggesting multiple, independent sources of acquisition. Studies from Europe and the United States similarly demonstrated considerable clonal variability and the dynamic nature of *S. aureus* infections (Masoud-Landgraf et al., 2015; Garbacz et al., 2018; Long et al., 2021). In a multicenter study of 246 children with CF in the USA, the majority of isolates exhibited substantial genetic heterogeneity, which is consistent with our results (Garbacz et al., 2018). Comparable findings were also reported by Eguizábal et al. (2024) in Spain, where the *S. aureus* isolates represented multiple clonal complexes, and showed little clonal relatedness (Eguizábal et al., 2024).

In this study, *S. aureus* showed relatively high resistance to macrolides and lincosamides. Comparable high rates of resistant strains were reported by De Tomi et al. and Tkadlec et al. in CF patients from Italy and the Czech Republic (Tkadlec et al., 2015; De Tomi et al., 2022). Azithromycin plays a special role in CF therapy, as it is frequently administered long-term at low doses for its immunomodulatory and anti-inflammatory effects. The higher proportion of macrolide-resistant strains observed in adolescents may potentially be related to cumulative antibiotic exposure, including repeated infections and prolonged or repeated courses of macrolides, which can promote the selection of resistant strains. However, detailed data on prior antibiotic use were not available in our study. Therefore, this possible association should be interpreted with caution.

Among *S. aureus* isolates, particularly those with the inducible MLSB phenotype, exposure to macrolides such as azithromycin can trigger the development of resistance, thereby reducing therapeutic efficacy and increasing the risk of treatment failure. The high prevalence of resistant strains observed in our study may thus reflect selective pressure favoring the emergence and persistence of resistant *S. aureus* strains. Consistently, a high proportion of isolates exhibited the MLSB resistance phenotype, with more than half of the isolates resistant to both macrolides and lincosamides. Notably, higher resistance rates were observed in older children. Therefore, as reported by Lee et al., the benefits and risks of continued therapy should be considered individually for each patient (Lee et al., 2021).

Although MRSA accounted for a relatively small proportion of isolates in our study, its presence remains clinically significant. In individuals with cystic fibrosis, MRSA colonization has been linked to accelerated decline in lung function and worse clinical outcomes. Therefore, our findings highlight the need for regular monitoring and measures to prevent MRSA transmission in vulnerable population (Chmiel et al., 2014; Elborn, 2016). In this study, MRSA represented distinct clones in individual patients, suggesting that MRSA cases may have arisen from independent events rather than transmission of a single epidemic strain among children. Once again, although regarding small sample size (nine MRSA isolates per 165 all *S. aureus* isolates) the exclusive occurrence of MRSA in patients colonized by a single *spa* type suggests that methicillin-resistant *S. aureus* strains tend to establish persistent and stable colonization in their hosts. The lack of competition from other clones may suggest a potential adaptation of MRSA to long-term colonization; however, further data are needed to support this interpretation. Furthermore, MRSA was not found in patients colonized with multiple *spa* types, which may suggest a tendency toward monoclonal colonization; however, previous studies have associated such patterns primarily with antibiotic exposure rather than with MRSA itself (Westphal et al., 2020). It is also worth noting that MRSA was identified in only two of the four age groups studied (0–1 year and 6–11 years). The presence of MRSA in infants may indicate hospital-acquired colonization or the early acquisition of a resistant strain that subsequently becomes the dominant clone as the child grows. However, the number of infants in the study group was relatively small. Nonetheless, the occurrence of MRSA in any CF patient warrants heightened vigilance, as it may worsen prognosis and substantially limit therapeutic options.

Analysis of *spa* types in most children revealed colonization with a single *S. aureus spa* type, often represented by repeatedly isolated strains, some of which exhibited multidrug resistance. The dominant type can likely persist in the respiratory tract for years, despite repeated courses of antibiotics, highlighting its adaptability. Intensive anti-staphylococcal therapy in these patients may eliminate competing strains, allowing only the most resistant clone to survive and dominate. As stated by Rumpf et al., this clone may accumulate minor genetic changes, but it remains essentially the same strain (Rumpf et al., 2021). Our *spa* typing results demonstrated cases where persistent isolates differed only by a few repeat variations, rather than being replaced by an entirely new clone. This clonal stability, especially evident in older children, is consistent with reports of patient-specific clonality of MRSA isolates and adaptive changes during persistent *S. aureus* infection in CF airways (Schwartbeck, 2016; Gabryszewski et al., 2019).

Our analysis indicated that colonization with multiple *spa* types was observed in a subset of patients, as reflected by the detection of different *S. aureus* strains in sequential samples. This variability may be associated with the acquisition of new strains over time, particularly in the absence of a dominant clone. Not all these strains exhibited high levels of resistance; some remained susceptible to macrolides and other antibiotics. The absence of highly resistant clones, such as MRSA, was noted in this group; however, the underlying factors remain unclear (Lange et al., 2020; Rumpf et al., 2021).

From a clinical perspective, this finding emphasizes the importance to customizing treatment strategies based on the colonization profile of patients. For those with established resistant clones, more aggressive or alternative treatment regimens may be required, such as combination therapies targeted MRSA or other resistant strains. On the other hand, for patients colonized with newly emerging and more susceptible clones, early eradication strategies may offer a valuable opportunity to prevent the development of resistance (Vanderhelst et al., 2013; Cunningham et al., 2022).

One limitation of the study is the relatively small sample size, uneven distribution across age groups, and the single-center design. In addition, detailed clinical data were not available, and the use of *spa* typing alone did not allow differentiation between superinfection and clonal evolution. Nevertheless, the results are consistent with the general view that *S. aureus* colonizes patients with cystic fibrosis and provides new insights into this population. Future studies using whole-genome sequencing could enhance our understanding of the dynamics of *S. aureus* clones in the respiratory tracts of CF patients. Despite these limitations, our findings provide valuable and clinically relevant insights into *S. aureus* colonization in this vulnerable patient population.

## 5 Conclusions

In conclusion, this study shows that *S. aureus* colonization in pediatric CF patients is largely patient-specific, with more than half of cases demonstrating monoclonal patterns and limited evidence of clonal transmission. Monoclonal colonization may be associated with the presence of MRSA, which is clinically relevant due to limited treatment options and the risk of more severe infections. A high frequency of resistance to macrolides and lincosamides, particularly in older children, may reflect cumulative antimicrobial exposure, although this could not be directly assessed. These findings highlight the importance of regular microbiological surveillance and careful use of antibiotics, especially long-term macrolide therapy.

## Data Availability

The raw data supporting the conclusions of this article will be made available by the authors, without undue reservation.
