# Supplementary material for: Molecular epidemiology of Staphylococcus aureus in pediatric cystic fibrosis patients: a single-center study
Source: Front Cell Infect Microbiol. 2026 May 28;16:1808334. doi: 10.3389/fcimb.2026.1808334 (PMC13253403; doi:10.3389/fcimb.2026.1808334)
Supplement: Supplementary file 1 [file Table1.docx]

## Table 1. Distribution of patients by age group and *spa* types

| Age group | Number of patients with 1 *spa* type | Number of patients with ≥2 *spa* types | Total patients |
| --- | --- | --- | --- |
| 0-1 | 10 (90.9%) | 1 (9.1%) | 11 |
| 2-5 | 15 (68.2%) | 7 (31.8%) | 22 |
| 6-11 | 19 (67.9%) | 9 (32.1%) | 28 |
| 12-18 | 11 (52.4%) | 10 (47.6%) | 21 |
| total | 55 (67.1%) | 27 (32.9%) | 82 |

## Table 2. Number of isolated *S. aureus* by age group and *spa* types

| Age group | 1 *spa* type | ≥2 *spa* types | Number of isolates |
| --- | --- | --- | --- |
| 0-1 | 13 (81.2%) | 3 (18.8%) | 16 |
| 2-5 | 22 (56.4%) | 17 (43.6%) | 39 |
| 6-11 | 29 (45.3%) | 35 (54.7%) | 64 |
| 12-18 | 20 (43.5%) | 26 (56.5%) | 46 |
| total | 84 (50.9%) | 81 (49.1%) | 165 |

## Table 3. Antibiotic resistance of *S. aureus* isolated from patients colonized with single *spa* type

| Age group | penicillin | macrolides | lincosamides | fluoroquinolones | aminoglycosides | tetracyclines | SXT | MLSB | MRSA |
| --- | --- | --- | --- | --- | --- | --- | --- | --- | --- |
| 0-1 | 10 (76.9%) | 5 (38.5%) | 5 (38.5%) | 0 (0.0%) | 1 (7.7%) | 2 (15.4%) | 3 (23.1%) | 0 (0.0%) | 3 (23.1%) |
| 2-5 | 20 (90.9%) | 6 (27.3%) | 6 (27.3%) | 2 (9.1%) | 2 (9.1%) | 0 (0.0%) | 0 (0.0%) | 1 (4.5%) | 0 (0.0%) |
| 6-11 | 28 (96.6%) | 20 (69.0%) | 20 (69.0%) | 7 (24.1%) | 5 (17.2%) | 4 (13.8%) | 0 (0.0%) | 4 (13.8%) | 6 (20.7%) |
| 12-18 | 19 (95.0%) | 9 (45.0%) | 6 (30.0%) | 3 (15.0%) | 1 (5.0%) | 1 (5.0%) | 1 (5.0%) | 0 (0.0%) | 0 (0.0%) |
| Total | 77 (91.7%) | 40 (47.6%) | 37 (44.0%) | 12 (14.3%) | 9 (10.7%) | 7 (8.3%) | 4 (4.8%) | 5 (6.0%) | 9 (10.7%) |

## Table 4. Antibiotic resistance of *S. aureus* isolated from patients colonized with multiple *spa* types

| Age group | penicillin | macrolides | lincosamides | fluoroquinolones | aminoglycosides | tetracyclines | SXT | MLSB | MRSA |
| --- | --- | --- | --- | --- | --- | --- | --- | --- | --- |
| 0-1 | 3 (100.0%) | 0 (0.0%) | 0 (0.0%) | 0 (0.0%) | 0 (0.0%) | 0 (0.0%) | 0 (0.0%) | 0 (0.0%) | 0 (0.0%) |
| 2-5 | 17 (100.0%) | 4 (23.5%) | 4 (23.5%) | 0 (0.0%) | 1 (5.9%) | 0 (0.0%) | 0 (0.0%) | 2 (11.8%) | 0 (0.0%) |
| 6-11 | 33 (94.3%) | 24 (68.6%) | 18 (51.4%) | 10 (28.6%) | 4 (11.4%) | 3 (8.6%) | 0 (0.0%) | 5 (14.3%) | 0 (0.0%) |
| 12-18 | 26 (100.0%) | 22 (84.6%) | 22 (84.6%) | 2 (7.7%) | 3 (11.5%) | 3 (11.5%) | 0 (0.0%) | 7 (26.9%) | 0 (0.0%) |
| Total | 79 (97.5%) | 50 (61.7%) | 44 (54.3%) | 12 (14.8%) | 8 (9.9%) | 6 (7.4%) | 0 (0.0%) | 14 (17.3%) | 0 (0.0%) |
